# Colony level fitness analysis identifies a trade-off between population growth rate and dauer yield in *Caenorhabditis elegans*

**DOI:** 10.1186/s12862-024-02199-1

**Published:** 2024-01-25

**Authors:** Hannah Chapman, Kuei Ching Hsiung, Isadora Rawlinson, Evgeniy R. Galimov, David Gems

**Affiliations:** Institute of Healthy Ageing, and Research Department of Genetics, Evolution and Environment, University College London, Gower Street, London, WC1E 6BT UK

**Keywords:** Adaptive death, *Caenorhabditis elegans* ecology, Evolution of aging, Inclusive fitness, Trade-off

## Abstract

**Background:**

In the evolution from unicellular to multicellular life forms, natural selection favored reduced cell proliferation and even programmed cell death if this increased organismal fitness. Could reduced individual fertility or even programmed organismal death similarly increase the fitness of colonies of closely-related metazoan organisms? This possibility is at least consistent with evolutionary theory, and has been supported by computer modelling. *Caenorhabditis elegans* has a boom and bust life history, where populations of nematodes that are sometimes near clonal subsist on and consume food patches, and then generate dauer larva dispersal propagules. A recent study of an in silico model of *C. elegans* predicted that one determinant of colony fitness (measured as dauer yield) is minimization of futile food consumption (i.e. that which does not contribute to dauer yield). One way to achieve this is to optimize colony population structure by adjustment of individual fertility.

**Results:**

Here we describe development of a *C. elegans* colony fitness assay, and its use to investigate the effect of altering population structure on colony fitness after population bust. Fitness metrics measured were speed of dauer production, and dauer yield, an indirect measure of efficiency of resource utilization (i.e. conversion of food into dauers). We find that with increasing founder number, speed of dauer production increases (due to earlier bust) but dauer yield rises and falls. In addition, some dauer recovery was detected soon after the post-colony bust peak of dauer yield, suggesting possible bet hedging among dauers.

**Conclusions:**

These results suggest the presence of a fitness trade-off at colony level between speed and efficiency of resource utilization in *C. elegans*. They also provide indirect evidence that population structure is a determinant of colony level fitness, potentially by affecting level of futile food consumption.

**Supplementary Information:**

The online version contains supplementary material available at 10.1186/s12862-024-02199-1.

## Background

Aging (senescence) is a largely genetically-determined phenomenon involving reduced fertility and increased mortality with increasing age. According to established theory it evolves due to the declining force of natural selection, leading to accumulation in populations of alleles with deleterious effects in later life, which may or may not also promote fitness earlier in life (antagonistic pleiotropy, AP) [1–3]. Yet certain features of aging in the nematode *Caenorhabditis elegans* suggest possible limitations to this account.

One such relates to reproductive senescence in this organism. *C. elegans* appear to actively limit their own reproduction, as follows. *C. elegans* hermaphrodites can reproduce either by self-fertilization or by mating with males. Given self-fertilization, reproductive span is limited by self-sperm number (~ 300), depletion of which inevitably leads to cessation of reproduction by around day 4 of adulthood [4]. Reproductive span is increased by mating with males thanks to increased sperm number. However, even in mated hermaphrodites replete with male sperm, visible deterioration and atrophy of the gonad occurs, and fertility drops rapidly with age [5]. How might such a seemingly fitness-destroying trait evolve? While the evolution of aging can be understood as the result of trade-offs between early life fitness benefits (particularly with respect to reproduction) and organismal senescence that reduces lifespan [2, 6], this high degree of curtailment of reproduction in *C. elegans* is an oddity [5, 7].

Another unusual feature of *C. elegans* aging is the ease with which its lifespan can be extended by mutation, particularly affecting insulin/IGF-1 signaling (IIS) [8]. A standard explanation is that the life-shortening effects of wild-type IIS reflect the presence of AP, i.e. that they must be coupled to some form of individual fitness benefit [9]. While this explanation could in principle be sufficient, it is notable that the magnitude of relative increases in *C. elegans* lifespan seen due to various interventions are far larger than those seen in other animals models, up to 10-fold in the case of IIS [10], and larger than those predicted by the AP theory [11].

We recently suggested a possible explanation for these peculiarities as follows. In the wild *C. elegans* exist largely as selfing, often near clonal populations of hermaphrodites on spatially restricted, ephemeral food patches (particularly rotting plant stems and fruit) [12]. Given their spatially discrete, viscous and clonal nature, such populations are comparable with colonies of unicellular microbes such as yeast and bacteria; we therefore, and for convenience, refer to them here as *C. elegans* colonies.

**Fig. 1:**
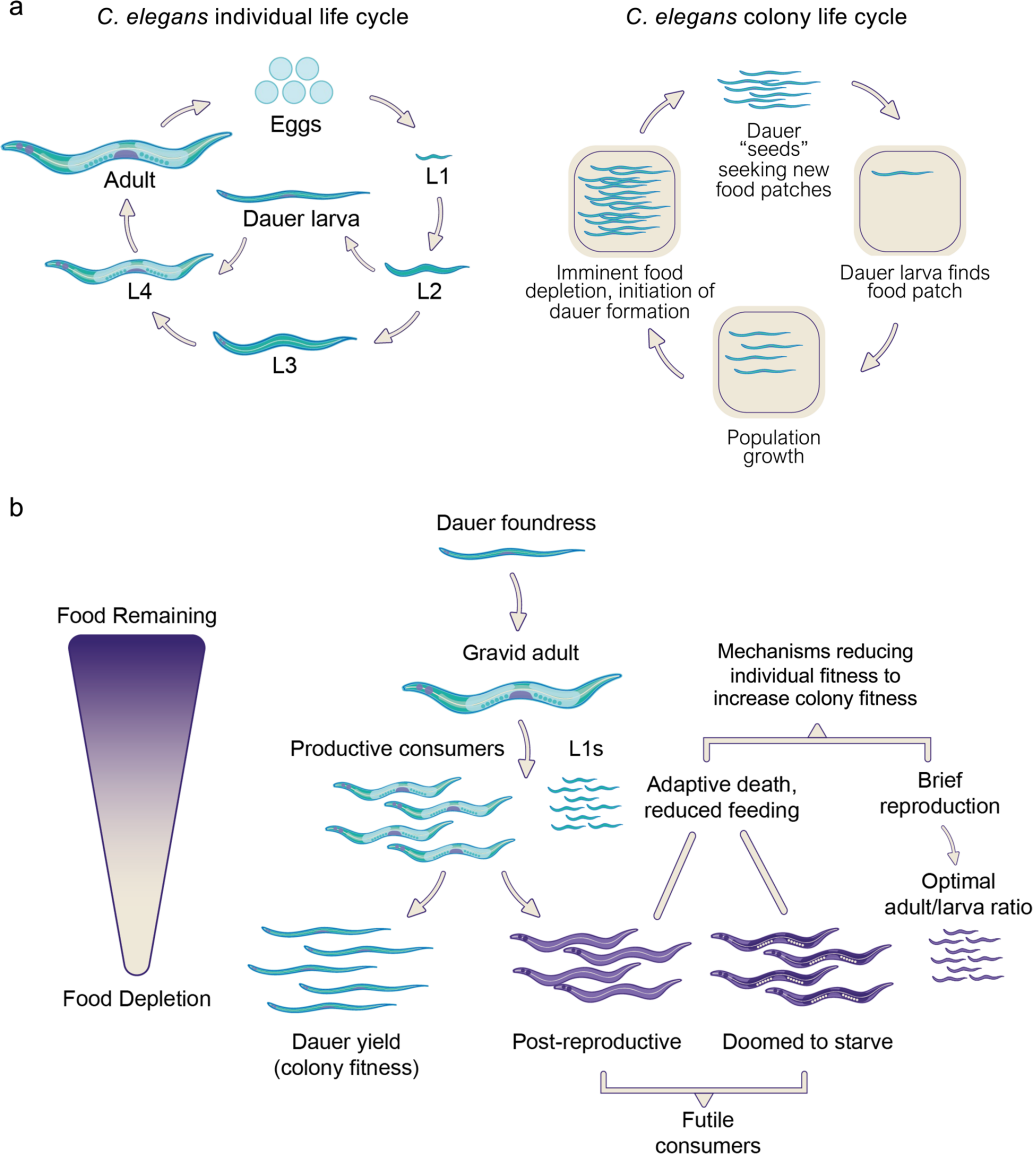
Natural selection may act on *C. elegans* fitness at the individual and the colony levels. **a** Left: the *C. elegans* individual life cycle. Under favorable conditions, the life cycle takes approximately 3 days. Hatching larvae develop into adults via four larval stages. Under adverse environmental conditions, *C. elegans* can developmentally arrest as a stress resistant alternative L3 stage, the dauer larva. Right: the *C. elegans* colony life cycle. Dauer larvae encountering a new food patch establish a new colony, for which production of dauer propagules is a measure of *colony* fitness (right), just as egg production by individual nematodes is a measure of *individual* fitness. **b** Colony fitness is optimized by maximizing efficiency of food conversion into dauers. According to this hypothetical scheme, post-reproductive mothers undergo programmed death to minimize futile food consumption, i.e., that which does not contribute to colony fitness but only to individual fitness. A short reproductive period may contribute to optimization of population structure to reduce numbers of larvae at a stage too early or late to form dauers. Image adapted from an earlier computer modelling study [18]

Such worm colonies exhibit a boom-and-bust life cycle [13]. After population growth, dwindling food and high population density triggers entry into the diapausal dauer larva stage, which is adapted for dispersal [14]. When dauer larvae encounter a new food patch, they can develop into adults and establish a new colony. Thus, *C. elegans* has life cycles at two levels: that of the individual nematode and that of the colony [15–17] (Fig. 1a).

The presence of two life cycles raises important questions relating to the nature of *C. elegans* evolution and how traits in individual nematodes affect fitness. Selection acts both within groups and between groups, which means that fitness has both a within-group and a between-group component. In *C. elegans* the former will be a function of age-specific reproductive rate within the food patch (individual fitness) [6, 19], and the latter a function of how likely a genotype is to leave a dauer descendent that makes it to the next patch (colony level fitness). This suggests that *C. elegans* is subject to multilevel selection (MLS) acting simultaneously at individual and colony levels [16, 20, 21]. More precisely, two forms of MLS may be distinguished as follows. MLS-1 relates to affects of natural selection on the frequency of individuals within a group, while MLS-2 considers differential survival and reproduction of groups in a meta-population of groups [20]. As an example, differential reproduction and survival of honey bee colonies may be understood in terms of MLS-2.

We previously suggested that fitness traits at the individual and colony level may trade off against each other, specifically that reductions in individual fertility and lifespan can increase colony fitness (in terms of dauer yield per colony) [16, 18, 22, 23]. According to this view, *C. elegans* evolution is shaped by selection at both the individual and colony level. It was previously noted that species which exploit ephemeral food sources often follow metapopulation dynamics [24]. Thus, programmed reproductive decline and organismal death can increase colony level fitness (or, viewed in another way, inclusive fitness) [25, 26] though it reduces individual fitness. In a similar fashion, in multicellular organisms, programmed cell death (apoptosis) and limitation of cell proliferation reduce fitness from the point of view of the individual cell, but can increase organismal fitness, and hence are often favored by natural selection [27, 28].

How can early death and reduced fertility in *C. elegans* increase colony fitness? We previously reasoned that early death might increase colony fitness through reducing food consumption by post-reproductive hermaphrodites that are post-reproductive (sperm-depleted as a consequence of protandry), thus increasing food availability for their younger kin. In other words, programmed, adaptive death promoting fitness via a consumer sacrifice mechanism [16, 23]. Previously, to test this hypothesis, we created an in silico model of *C. elegans* colonies, each subsisting on a virtual food patch. Colony fitness was measured as dauer yield, and compared between colonies with virtual worms with different life history traits. Simulations showed that under some conditions shorter life did indeed reduce the proportion of food consumed by adults, and increase colony fitness [18]. These findings were broadly consistent with earlier computer simulations of a more generic nature (i.e. that did not model any specific species) [29–32].

Unexpectedly, in the virtual *C. elegans*, where daily fertility was high, virtual worms with shorter reproductive spans led to higher colony fitness. How might this occur? One possibility is that it reflects the importance of population structure (i.e. relative proportions of different larval and adult stages, not the genetic structure) as a determinant of colony structure. In terms of maximal fitness, an ideal population structure would be one in which all consumption of the *E. coli* food source contributed ultimately to dauer yield. However, as the behavior of the model suggested, there are several forms of non-productive, futile food consumption: consumption by post-reproductive hermaphrodites at any time, and consumption immediately prior to food exhaustion by all stages except early larvae destined to become dauers. Thus, an ideal population structure is one that maximizes the number of pre-dauer larvae just as food is depleted, and minimizes all other stages (Fig. 1b). We hypothesized that *C. elegans* curtail their reproduction in order to optimize population structure and colony fitness [18, 22].

In this study, we have begun to probe predictions arising from computer modelling regarding the effect of population structure on fitness by testing effects of altering population structure on colony fitness in real *C. elegans*. Our results provide evidence of the importance of population structure as a determinant of *C. elegans* fitness, and of the presence of trade-offs between individual and colony fitness, consistent with superorganismal features of *C. elegans* colonies. They also suggest the presence of a trade-off between two colony fitness traits: speed of dauer production and dauer yield.

## Results

### Food patch consumption rate increases with founder number

**Fig. 2:**
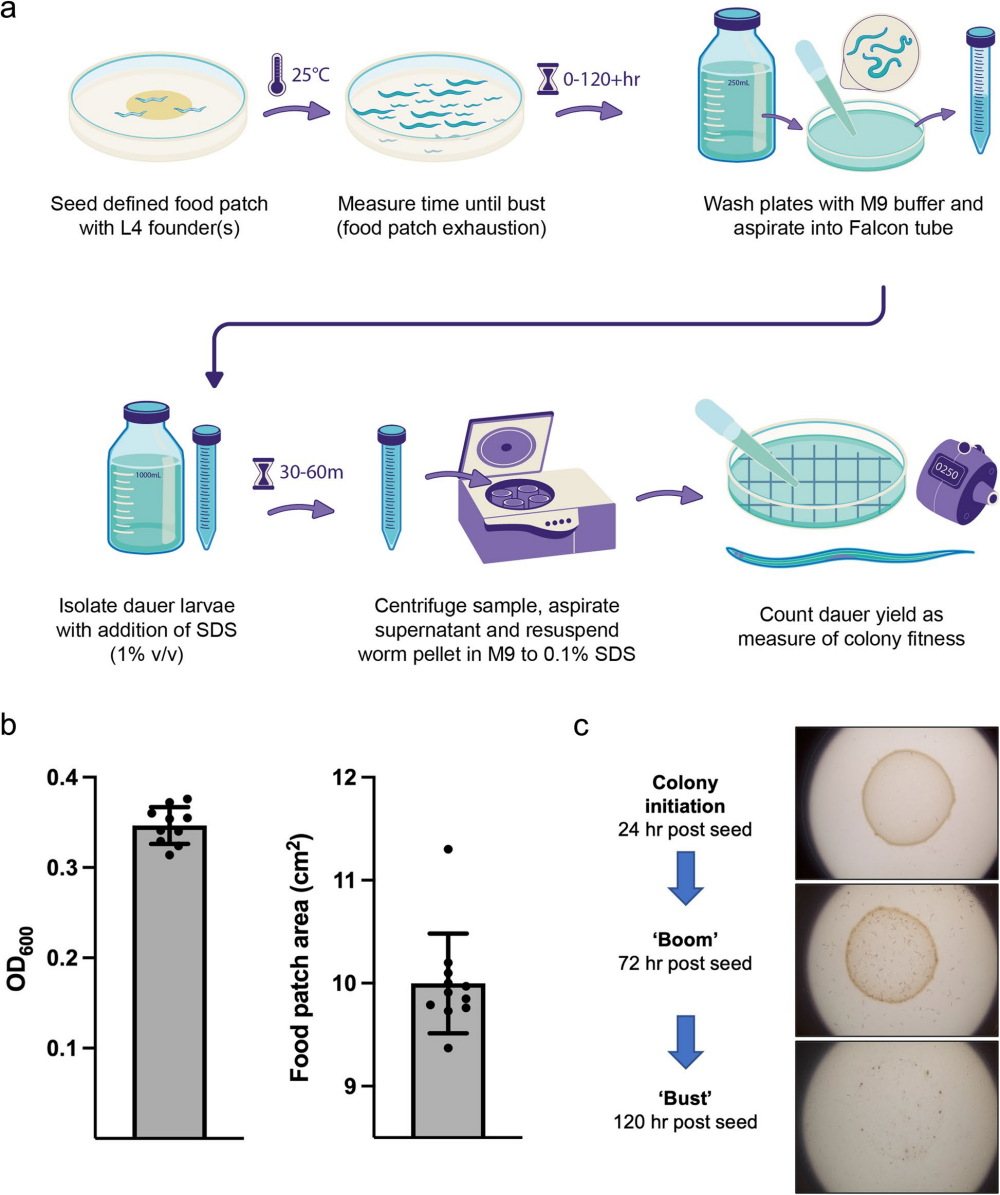
The colony fitness assay. **a** Measurement of dauer yield (colony fertility). **b** Assessment of variation in food patch size; the degree of variation is modest. Mean ± S.D. (*n* = 10).** c** Illustration of colony appearance at boom and bust. The latter is distinguished by absence of the *E. coli* lawn, and the dispersal of worms across the entire agar plate [33]

In this study, we explore the possibility that colony fitness is influenced by colony population structure. Two measures of colony fitness are resource utilization speed and efficiency, the latter measurable as dauer yield (effectively, *colony fertility*). To vary population structure, we established colonies with 1, 3, 10, 50 or 200 founder nematodes (Fig. 2a).

For colony fitness assays an invariant, constant food patch size was required. In a test verification of food patch constancy, bacterial lawn area, and optical density of resuspended lawns were measured (*n* = 10), and found to vary over only a narrow range, from 9.3 to 11.3 cm^2^, mean 9.9 ± 0.48 cm^2^ (S.D.) and OD_600_ from to 0.314 to 0.376, mean 0.346 ± 0.020 (S.D.) (Fig. 2b). Raw data for Fig. 2b is available in Supplementary dataset 1.

Nematode populations were allowed to grow until they had consumed all of the available food (transitioned from boom to bust stage) (Fig. 2c), and dauer larva number was then measured at a set time after bust. A previous study found that at 22 °C eggs laid on a crowded plate close to food depletion developed into dauer larvae after ~ 40 hr. [14]. To attempt to estimate the entire dauer yield, we selected the later time point of 72 hr.

**Fig. 3:**
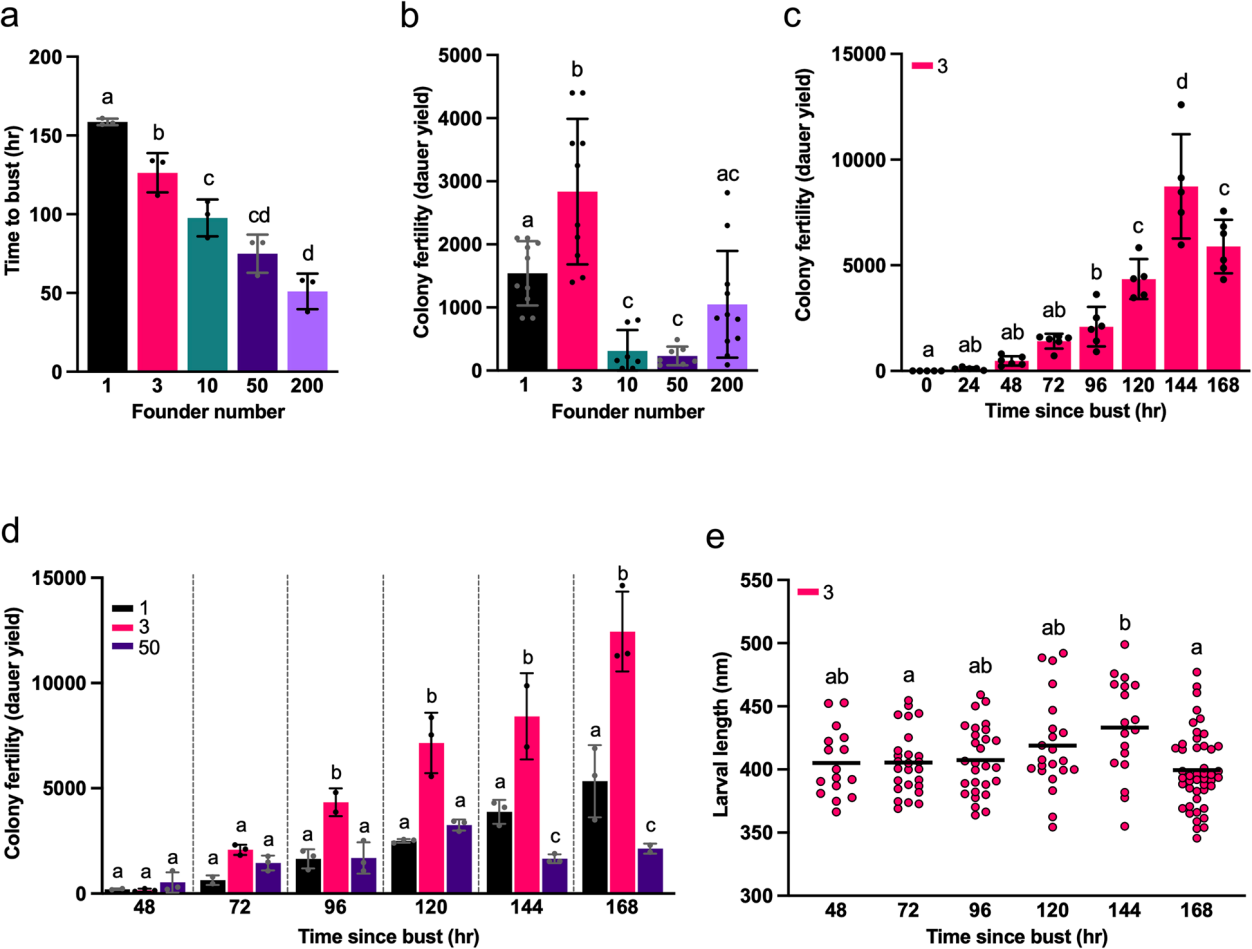
Effects of founder number on colony fitness metrics and population structure. **a** Time to bust decreases with increasing founder number. Means ± S.D. of 3 biological replicates. One-way ANOVA with Tukey’s multiple comparisons test. 1 vs 3, *p* = 0.0260; 1 vs 10, *p* = 0.0003; 1 vs 50, *p* < 0.0001; 1 vs 200, *p* < 0.0001; 3 vs 10, *p* = 0.0500; 3 vs 50, *p* = 0.011; 3 vs 200, *p* = < 0.0001; 10 vs 50, *p* = 0.1432 ns; 10 vs 200, *p* = 0.0023; 50 vs 200, *p* = 0.1139 ns. **b** Dauer yield (72 hr post bust) is highest in populations started with 3 founders. Means ± S.D. of at least 7 biological replicates. 3 vs 1, *p* = 0.0033; 3 vs 10, *p* = < 0.0001; 3 vs 50, *p* < 0.0001; 3 vs 200, *p* < 0.0001. **c** Dauer yield peaks at 144 hr after population bust (3 founders, 25 °C). Means ± S.D. of at least 5 biological replicates. **d** Dauer yield is greatest given 3 founders. Estimates of dauer yield given 1, 3 or 50 founders up to 168 hr after population bust. Means ± S.D. of 3 biological replicates. Two-way ANOVA with Tukey’s multiple comparisons test. 96 hr: 1 vs 3, *p* = 0.0073; 3 vs 50, *p* = 0.0083. 120 hr: 1 vs 3, *p* < 0.0001; 3 vs 50, *p* < 0.0001. 144 hr: 1 vs 3, *p* < 0.0001; 1 vs 50, *p* = 0.0135; 3 vs 50, *p* < 0.0001. 168 hr: 1 vs 3, *p* < 0.0001; 1 vs 50, *p* = 0.0014; 3 vs 50, *p* < 0.0001. **e** Increase in dauer length at 144 hr post bust. Line at mean (*n* > 15 worms per time point). One-way ANOVA with Tukey’s multiple comparisons test. Plots marked with different letters are significantly different from each other. Raw data for Fig. 3a-e is available in Supplementary dataset 1

To measure resource utilization speed, and to facilitate identification of the 72 hr post-bust time point, we first measured time from colony foundation to bust for each number of founders. This showed that time to bust decreased with increasing founder number. Time to bust varied over a ~ 3-fold range, from 51 hr given 200 founders to 159 hr given 1 founder (Fig. 3a). This is unsurprising, since a larger founder number is expected to lead to faster population growth and earlier bust. That a larger founder number leads to earlier bust and dauer formation implies that larger founder number provides a potential colony fitness benefit, in the form of faster colony level reproduction.

### Dauer yield is affected by founder number

Next, dauer yield was measured at 72 hr post bust. Dauer yields showed considerable variation between trials throughout these studies, suggesting that this measure of fitness is highly sensitive to subtle differences in culture conditions and/or susceptible to random variation. For example, dauer yields given 3 founders ranged from 1400 to 4400 (Fig. 3b).

Despite this, significant effects of founder number on dauer yield were detected. Firstly, colonies derived from 3 founders yielded significantly more dauers than those derived from 1, 10, 50 or 200 founders (*p* = 0.0033, < 0.0001, < 0.0001 and < 0.0001, respectively). Secondly, colonies established from 1 founder yielded more dauers than those from 10 and 50 founders (*p* = 0.0146, 0.0083, respectively). Dauer numbers did not differ significantly between colonies derived from 10, 50 and 200 founders. Mean dauer yield given 3 founders was 2837 (1400-4400), compared to 1540 (830–2100), 310 (37–800), 232 (83–490) and 1050 (90–2820) given 1, 10, 50 and 200 founders, respectively.

The observation that founder number affects dauer yield led us to examine this effect in more detail. One concern was that the 72 hr post-bust time point might be before or after the peak in dauer yield. A previous study reported that at the lower temperature of 20 °C peak dauer numbers were not reached until 144–168 hr (6–7 days) post bust [34]. To address this, we tested effects of varying founder number on dauer yield over a similar time frame.

We first measured dauer yield from 3 founders daily up to 168 hr post bust (25 °C). Dauers began to appear from 48 hr, reaching peak numbers at 144 hr (Fig. 3c). Next, daily dauer yield up to 168 hr post bust was compared with 1, 3 and 50 founders. Again, dauer yield from a colony established from 3 founders was significantly higher from 96 hr onwards than that from 1 founder and 50 founders (Fig. 3d) (96 hr: 1 vs 3, *p* = 0.0073; 3 vs 50, *p* = 0.0083. 120 hr: 1 vs 3, *p* < 0.0001; 3 vs 50, *p* < 0.0001. 144 hr: 1 vs 3, *p* < 0.0001; 1 vs 50, *p* = 0.0135; 3 vs 50, *p* < 0.0001. 168 hr: 1 vs 3, *p* < 0.0001; 1 vs 50, *p* = 0.0014; 3 vs 50, *p* < 0.0001, two-way ANOVA with Tukey’s multiple comparisons test). Taken together these findings show that the optimal founder number differs for speed of dauer production and dauer yield, demonstrating the presence of a trade-off between these two fitness traits. The relationship between founder number and colony fertility (dauer yield) is consistent with an effect of population structure on colony level fitness.

### Early recovery of some dauers after population bust

Dauer diapause can endure for up to several months [35]. However, there was a significant drop in dauer number at later time points (144 vs. 168 hr, *p* = 0.0018) (Fig. 3e). One possibility is that this reflects increased burrowing of dauers into the agar, such that they were not recovered during dauer assays. However, examination of the agar in selected plates revealed very few burrowed dauers. Moreover, dauer yield was not increased on harder, 2.5% agar NGM plates (expected to reduce burrowing). Thus, the decline in dauer number is unlikely to be due to burrowing. A second possibility (not excluded) is that dauers escaped from the agar surface onto the walls and lids of the plates.

A third possibility is that some dauers recover from diapause. Consistent with this, from 120 hr after population bust onwards, some signs of dauer recovery were observed, including increased body length (Fig. 3e) and pharyngeal pumping. This suggests that some individuals may exit dauer arrest relatively soon after bust in the wild.

### Modelling-based illustration of effects of founder number on population structure

We reasoned that the population structure of *C. elegans* colonies will exhibit complex, time-dependent changes that are affected by founder number. To obtain a clearer picture of the likely nature of such complex changes, we modelled them using a computer simulation. To do this, a transition matrix was built using the known rate of wild-type *C. elegans* development and timing of egg-laying (egg-laying dynamics) at 25 °C [36].

To model the laboratory tests performed, a virtual food patch of 10 million food units was provided to the numbers of L4 founders used in our prior tests (1, 3, 10, 50, 200). In the model, food consumption rate per hour was one unit for L1, two for L2, four for L3, eight for L4 and 16 for adults. Population structure at 1 hr intervals was estimated using a matrix population model over a 200 hr period. This allows comparisons to be made of population structure at the time of bust with different founder numbers. Such predictions are expected to be highly approximate, particularly since effects of crowding and dwindling food stocks prior to bust are not considered. Nevertheless, the modelling allows several plausible predictions to be made.

**Fig. 4:**
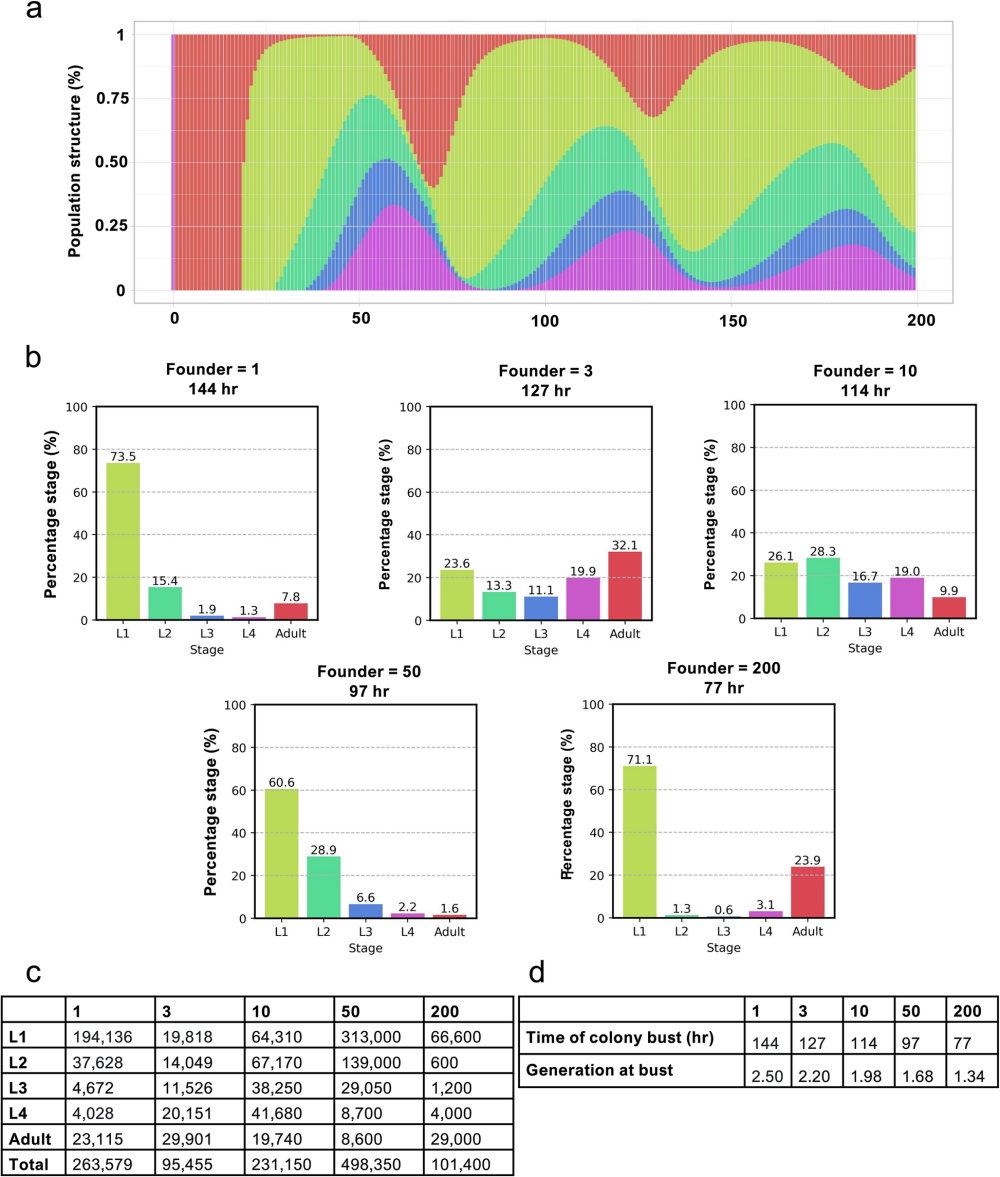
Complex variation in population structure in virtual *C. elegans* colonies. **a** Population structure of virtual *C. elegans* over 200 hr beginning at the final hour of the L4 stage. **b** Founder number affects proportions of developmental stages at bust. **c** Relative population structure at bust. Total number of larval and adult stages within a population at bust for each founder. Percentages are show in Fig. 4b. **d** How founder number is predicted to affect the number of generations traversed at the time of bust. Output from computer simulations, based on previously reported developmental timings at 25 °C [36]. Generation 1 is P_0_, 2 F_*1*_ and 3 F_*2*_

First, as expected, colony population structure changes over time in a complex manner, with major changes in the relative frequency of different developmental stages (Fig. 4a). Second, at the time of bust, the relative proportions of different developmental stages differ between populations with different founder numbers (Fig. 4b, c). Third, the modelling suggests that given lower founder numbers (1, 3, 10) dauers develop from L1s of the F2 generation, while given higher founder numbers (50, 200) they form largely from L1s of the F1 generation (Fig. 4d).

In its prediction of the effects of founder number on time to bust (Fig. 4d), the model is consistent with the experimental data (Fig. 3a). However, this is not the case with respect to effect of founder number on dauer yield. In terms of the number of L1 and L2 larvae at bust, 50 founders are predicted to give the largest yield, and 3 founders the smallest. By contrast, experimental data shows that 3 founders gives the largest dauer yield (Fig. 3b, d). This likely reflects limitations of the model in accurately reflecting population dynamics and dauer larva formation.

## Discussion

This report describes a first pass, preliminary exploration of *C. elegans* colony fitness, and how it may be affected by population structure. The finding that colony fertility (dauer yield) rises and falls with increasing founder number is consistent with the prediction, based on computer simulations and evolutionary theory [16, 18], that colony population structure is a determinant of colony fitness. That one colony fitness trait, speed of resource utilization, increases with increasing founder number, while another, colony fertility (dauer yield), rises and falls with increasing founder number, defines a trade-off at colony level between resource utilization speed and efficiency. One way in which optimal founder number could maximize dauer yield is by generating a population structure that minimizes futile food consumption (i.e. that which does not contribute to dauer yield) [18]. Our computer simulations of *C. elegans* populations provide an indication of the complexity that is present in colony development. Together, these forays into a new topic, the biology of *C. elegans* colony fitness, provide a starting point for future studies of actual population structural dynamics, including analysis of colony efficiency at converting food into dauer larvae.

### Evidence that population structure optimization improves fitness by minimizing futile food consumption

Previous theoretical arguments, supported by computer modelling of *C. elegans* colonies, support the prediction that trade-offs occur between fitness at the individual and colony levels [16, 18, 22]. Several possible adaptations could reduce futile food consumption, including programmed organismal death (adaptive death) of post-reproductive animals, decline with age in food consumption rate [37] and reduction of reproductive span in order to assure a population structure that is optimal in terms of minimizing futile food consumption. Such traits represent *costly programs* that harm the *C. elegans* individual but benefit the *C. elegans* colony [38].

The present study provides support for the view that minimizing futile food consumption promotes colony fitness. We show that dauer yield varies with founder number, in a manner that implies that dauer yield is affected by colony structure. Behavior of virtual *C. elegans* colonies implies that, under some conditions, reducing fertility of individual nematodes could increase colony fitness, as measured by dauer yield [18].

Under the conditions tested, 3 founders was optimal for maximizing colony fertility. This is broadly consistent with the recent estimation that wild *Caenorhabditis* colonies from selected tropical ecologies were derived from a maximum of 3–10 founders [39], in line with earlier observation from temperate climates [12]. However, one might expect that the number of founders that optimizes colony fertility would be condition dependent, particularly with respect to food patch size (e.g. more founders for a larger food patch). This remains unexplored.

An optimal founder number leads to the prediction that lower fertility may increase colony fertility (dauer yield) where founder number is too high and, conversely, higher fertility may increase colony fertility where founder number is too low. We did attempt to test this using *fem-3(e1999)* and *tra-3(e2333)* mutants, which have reduced and increased brood size, respectively [33], but results were inconclusive, and uninterpretable without analysis of actual effects on population structure. Thus, these predictions remain to be proven or disproven.

### Why is development of starvation dauers slower than that of constitutive dauers?

It was previously observed that dauers appear relatively late after population bust: accumulating from day 3 after bust, to peak numbers on days 6–7 (20 °C, single L4 founder) [34]. Our results are consistent with this, and the deduction that dauer yield arises largely from pre-dauer larvae that continue development despite the paucity of food in plates post bust.

We initially reasoned that a very large founder number would lead to so many first stage (L1) larvae that the entire food patch would be consumed before any dauer larvae could form, leading to a colony fertility of nil. However, with even 200 founders, expected to produce approximately 34,000 F1 progeny over the span of 5 reproductive days, many dauers were produced.

Our results imply that under bust conditions, pre-dauer larvae complete dauer development despite very low food availability. How do they manage this? One possibility is that decomposing corpses of starved adults and larvae provides nourishment, supporting development. Another is nourishment from the contents of the NGM agar.

The slow pace of development in starvation dauers relative to that in constitutive dauers is likely attributable to nutrient insufficiency, either acting directly on growth, or via nutrient sensitive growth regulatory pathways (e.g. insulin/IGF-1 and TGF-β signaling).

### Dauers formed in a colony recover more quickly than induced dauers

Dauer larvae form in response to high temperature, low food and high levels of dauer pheromone, and recover and resume development when these conditions are reversed [40]. We therefore did not expect to see recovery of dauers on the starved plates studied in our assays. However, some recovering dauers were seen (Fig. 3e), perhaps reflecting dauer behavior in wild, depleted colonies. One possibility is that this reflects a bet hedging strategy, where some dauers risk premature exit from diapause in the hope of being to be the first to colonize new food patches.

## Conclusions

The findings presented here begin to reveal a relatively unexplored aspect of *C. elegans* biology that warrants further investigation: the superorganism-like properties of colonies. Of particular interest for future research will be analysis of actual population structure, and how it affects efficiency of conversion of food biomass into dauer biomass. This is important for understanding the fundamental biology of this intensively studied animal model, and also as a means to investigate the evolution of altruistic behavior and aging in colonial organisms.

## Methods

### Strains and maintenance

*C. elegans* populations were maintained using standard protocols [41] on Nematode Growth Media (NGM) agar plates (60 mm diameter) with *Escherichia coli* strain OP50 as a food source, and incubated at either 20 °C or 25 °C. All tests were performed with the *C. elegans* N2 hermaphrodite stock (N2H) [42].

### Computational prediction of population structure

Population structure at a series of time intervals and at the time of bust, given different founder numbers and at 25 °C was calculated from previous measurements of development time [36]. The code for simulating population structure is available at https://github.com/KCHsiung/C.-elegans-population-dynamic.git. Python (3.9.5) was used to implement the model with the addition of the following data analysis with libraries: pandas (1.4.1) and numpy (1.22.3); data visualisation with libraries: matplotlib (3.5.3) and plotnine (0.9.0).

### Measurement of dauer yield (colony fertility)

Nematode populations were established using late stage L4 larvae raised at 20 °C, and then maintained at 25 °C, at which temperature dauer larva formation occurs more readily [40]. Lawn size was kept approximately constant by adding a fixed volume of *E. coli* suspension and allowing the bacterial lawn to grow to reach its maximum thickness. *E. coli* OP50 culture medium (0.5% (w/v) Bacto Tryptone (BD Difco) and 0.25% (w/v) yeast extract (BD Difco) in MilliQ water was inoculated with a single OP50 colony and grown for 16–18 hr at 37 °C with shaking until reaching an OD_600_ of 1. NGM plates were then seeded with bacterial suspension and incubated at 37 °C for 24 hr.

To prepare synchronized nematodes for colony foundation, gravid N2 hermaphrodites were placed on NGM plates for up to 3 hr to lay eggs and then removed. After 48 hr at 20 °C, late stage L4 worms were picked onto food patch plates, which were then sealed with parafilm to reduce plate drying and avoid escape of worms (particularly dauers), and incubated at 25 °C. For all trials, a minimum of 3 plates were set up for each founder number and each time point.

Dauer larvae possess a thickened cuticle and sealed buccal cavity [14], which makes them resistant to exposure to 1% sodium dodecyl sulfate (SDS). To identify dauer larvae, worms were washed from the plate surface, pelleted by centrifugation at 2000 RPM for 2 min, and resuspended in 1% SDS. After incubation at room temperature for 30–60 min, SDS was removed and worms resuspended in M9. Dauer number was estimated by pipetting 1 ml of sample onto a non-seeded NGM plate and counting them. Three counts per sample were taken, from which an average was calculated. Counting accuracy was aided by placing beneath the plate a Petri plate lid on which a 5 × 5 grid had been drawn.

### Determination of resource utilization speed

After the addition of founders and incubation at 25 °C, plates were monitored every 6–12 hr until food patch exhaustion (population bust), which was recognizable by the disappearance of the bacterial lawn and dispersal of worms across the plate [33] (Fig. 2c).

### Estimation of number of burrowed dauer larvae

Agar from several NGM plates with bust populations was cut into small cubes using a scalpel, and placed in M9 buffer in a Falcon tube, and left on a rotator for 2–3 hr (room temperature). The agar cubes were then removed, SDS added to 1%, and dauers counted.

### Identification of recovering dauer larvae

A sample of 1% SDS-treated nematodes was placed on 2% agar pads and anaesthetized in a drop of 0.2% levamisole, and a cover slip placed on top. Images were captured using a Zeiss ApoTome.2 microscope with a Hamamatsu digital camera C13440 ORCA-Flash4.0 V3 and Zen software. Recovering dauers were identified by virtue of one or more of the following characteristics: increased diameter of the pharynx, twitching in the terminal bulb of the pharynx (recovery of pharyngeal pumping function) and increased body length.

### Data analysis and statistical tests

Graphs were created and statistical analysis was carried out using the application GraphPad Prism (9.3.1). Dauer yields were compared using an ordinary one-way ANOVA with Dunnett’s multiple comparisons test or Tukey’s multiple comparisons test unless specified. Data from culture plates on which bacterial or fungal contamination was detected were excluded from further analysis.

### Supplementary Information

**Additional file 1. Supplemental Dataset 1.** Raw data corresponding to parts of Figures 2 and 3.

## Data Availability

The datasets used and/or analyzed during the current study are available from the corresponding author on request.
